# Mental health, serum biomarkers and survival in severe COPD: a pilot study

**DOI:** 10.1186/s40248-016-0041-8

**Published:** 2016-01-18

**Authors:** Christian Zilz, Stefan H. Blaas, Michael Pfeifer, Rudolf A. Jörres, Stephan Budweiser

**Affiliations:** 1 Department of Internal Medicine, HELIOS Hospital Munich-Perlach, Munich, Germany; 2 Center for Pneumology, Donaustauf Hospital, Donaustauf, Germany; 3 Institute and Outpatient Clinic for Occupational, Social and Environmental Medicine, Ludwig-Maximilian University, Munich, Germany; 4 Department of Internal Medicine II, Division of Respirology, University of Regensburg, Regensburg, Germany; 5 Department of Internal Medicine III, RoMed Clinical Center Rosenheim, Rosenheim, Germany

**Keywords:** Anxiety, Chronic respiratory disease, Depression, Mortality, Prognostic factors, Systemic inflammation

## Abstract

**Background:**

Chronic obstructive pulmonary disease (COPD) impairs physical status and impacts on mental health. This prospective study was designed to assess associations between mental health and systemic biomarkers, and their combined relationship with long-term survival in stable severe COPD.

**Methods:**

Forty-five patients with severe but stable COPD (forced expiratory volume in 1 s of 29.8 (quartiles: 22.6; 41.4) %predicted) were assessed using the Hospital Anxiety and Depression Scale (HADS), the Patient Health Questionnaire (PHQ), St. George’s Respiratory Questionnaire and the State-Trait Anxiety Inventory (STAI). The following serum biomarkers were measured: 25-OH-cholecalciferol, C-reactive protein, erythrocyte sedimentation rate, leucocyte number, serum amyloid-A (SA-A), N-terminal pro-brain natriuretic peptide, troponin I, glycosylated haemoglobin, haemoglobin (Hb), haematocrit (Hc), creatinine and thyroid-stimulating hormone. Patients were followed-up for 36 months. Associations between aspects of mental health and biomarkers, and their utility as predictors of 3-year survival were evaluated by regression analyses.

**Results:**

The prevalence of anxiety (HADS-A: 89.9 %), depression (HADS-D: 58.8 %; PHQ: 60.6 %), somatisation (PHQ-15: 81.8 %) and psychosocial stress (PHQ-stress: 60.6 %) was high. There was a significant positive association between the leucocyte count and SA-A level with STAI-trait anxiety (*p* = 0.03 and *p* = 0.005, respectively), and between leucocytes and PHQ-stress (*p* = 0.043). Hb and Hc were significantly negatively associated with HADS-depression (*p* = 0.041 and *p* = 0.031, respectively). Univariate Cox regression analyses revealed that leucocyte count (hazard ratio (HR) 2.976, 95 % CI 1.059-8.358; *p* = 0.038), and stress (HR 4.922, 95 % CI 1.06–22.848; *p* = 0.042) were linked to long-term survival. In multivariate Cox regression analyses, including known risk factors for survival in COPD, PHQ-stress (HR 45.63, 95 % CI 1.72–1,208.48; *p* = 0.022) remained significantly associated with survival.

**Conclusion:**

In this pilot study different dimensions of mental health were correlated to serum biomarkers, probably reflecting systemic effects of COPD. While leucocyte number and PHQ-stress were associated with long-term survival in univariate analyses, PHQ-stress remained in multivariate analyses as independent prognostic factor.

## Background

Chronic obstructive pulmonary disease (COPD) is a systemic disease whose multiple dimensions are increasingly uncovered by research. This has promoted the concept of clinical phenotypes, which attempts to identify specific subgroups of COPD patients with similar clinical characteristics, treatment needs and prognosis [1]. As a result, several clinical phenotypes, which can occur simultaneously, are assumed, e.g., patients with marked hyperinflation, frequent exacerbations, rapid FEV_1_ decline, metabolic and cardiovascular comorbidities, or eosinophilic inflammation [1].

Importantly, COPD not only impairs physical status but also has impacts on mental health [2–5]. Owing to the prevalence of mental health problems in COPD further evaluation is needed. Mental health involves various aspects, such as psychiatric issues, somatisation and disease-evoked psychological strain. Among these, depression and anxiety are strongly related to worse long-term outcome in COPD [6–8].

Similarly, health-related quality of life (HRQOL) is a broad multidimensional concept that usually includes self-reported measures of physical and mental health [9], and is also associated with long-term survival [10, 11] and acute exacerbation [12].

Unfortunately, assessing mental health is time consuming and complex, because either an interview with an experienced expert or complicated psychosocial assessments with questionnaires are necessary. Further problems lie in the quality of the completed questionnaires. Patients may have a poor compliance with regard to mental health assessment via questionnaires, perhaps due to misunderstanding, age-related diseases (e. g., cognitive or vision impairment) or lack of interest. Clinically desirable would be an alternative approach in assessing mental health, especially if it is independent from the patient’s active cooperation, as well as easily to obtain.

Serum biomarkers have been mostly explored in COPD for estimating the risks of exacerbation and for long-term monitoring [13], including procalcitonin [14], N-terminal pro-brain natriuretic peptide (NT-ProBNP) and troponin [15] for short-term assessment, and leukocyte number, fibrinogen and CRP in relation to long-term survival [16], as well as blood eosinophil count as biomarker for the response to inhaled steroids [17].

Due to its systemic involvement, associations with serum biomarkers could also occur regarding mental health, particularly in severe COPD. Indeed, associations between depression and the level of biomarkers, such as C-reactive protein (CRP) [18], haemoglobin (Hb) [19], thyroid-stimulating hormone (TSH) [20, 21] and vitamin D [22–25], have been reported.

Based on these considerations, it seems plausible to explore possible associations between mental health parameters and serum biomarkers. We therefore assessed whether in stable severe COPD different dimensions of mental health assessed via questionnaires correlated with established serum biomarkers and whether they were associated with long-term survival.

## Methods

### Study population

This prospective study included patients with COPD Global Initiative for Chronic Obstructive Lung Disease (GOLD) stages III and IV, aged 35–95 years, admitted to the Center for Pneumology at Donaustauf Hospital in a clinically stable state between October 2009 and October 2010. Patients with a moderate to severe exacerbation defined by a serum CRP level ≥20 mg/dl, a blood pH <7.35, or having received antibiotic therapy within the last 2 weeks were not included. Other exclusion criteria were acute pneumonia confirmed by X-ray; lung resection; thoracic deformations, or refusal to participate.

Eligible patients were enrolled at control appointments for non-invasive intermittent positive pressure ventilation or long-term oxygen therapy, and from new referrals to the clinic. Demographic data, blood samples, physical function, mMRC dyspnea scale, Cumulative Illness Rating Scale-Geriatric and mental health (questionnaires) were assessed after inclusion in the study; notification of death during the study period (at least 36 months for each patient after inclusion) was obtained from the patient’s relatives or family doctor by telephone.

### Blood samples

Peripheral blood samples were evaluated for the following: leucocyte count, Hb level and the haematocrit (Hc) (ABX Micros 60-CT analyser, equipped with v.1.0 software, Horiba ABX, Montpellier, France); creatinine, CRP and TSH levels (Dimension Xpand system, Dade Behring, Schwalbach, Germany); glycosylated haemoglobin (HbA1c; Cobas Integra 400 plus analyser, Roche Diagnostics Deutschland GmbH, Mannheim, Germany); 25-OH-cholecalciferol (25-OHD), serum amyloid-A (SA-A) and troponin I (ADVIA Centaur TnI-Ultra assays, Bayer Vital GmbH, Fernwald); NT-proBNP (Elecsys 2010 analyser, Roche Diagnostics GmbH); and the erythrocyte sedimentation rate (ESR).

### Functional analysis

Lung function assessment comprised post-bronchodilator spirometry and body plethysmography performed using a Masterlab system (Viasys Inc., Würzburg, Germany) according to the recommendations of the American Thoracic Society (ATS) [26]. The predicted values for forced expiratory volume in 1 s (FEV_1_) and vital capacity (VC) were calculated using the reference values determined by the European Community for Steel and Coal [27].

A six-minute walk distance (6-MWD) test was performed according to the recommendations of the ATS [28]. The predicted 6-MWD was calculated using age, gender, height and weight [29].

### Mental health

Aspects of mental health were measured using the German version of the Hospital Anxiety and Depression Scale (HADS) [30], the Patient Health Questionnaire (PHQ) [31], the St. George’s Respiratory Questionnaire (SGRQ) [32] and the State-Trait Anxiety Inventory (STAI) [33].

The HADS was originally developed as a screening tool for anxiety and depression in an outpatient setting but is now widely used in general hospital practice for non-psychiatric clinical populations [30, 34]. It is composed of a total of 14 questions on a questionnaire each scored on a four-point Likert scale (0–3); there are seven questions for symptoms of anxiety (HADS-A) and seven for symptoms of depression (HADS-D) each, therefore, with a total score range of 0–21 [30]. Higher scores indicate more severe symptoms [30]. The following categories (cut-off scores) for HADS-A and HADS-D were used: no (0–7), borderline (8–10), severe (11–14) and very severe (15–21).

The PHQ is a self-administered version of the Primary Care Evaluation of Mental Disorders (PRIME-MD) and is used in primary medical practice to determine psychosocial and somatic stress factors as well as to screen for the five most frequent psychiatric disease groups [31, 35]. The complete version comprises 78 questions to which answers are evaluated with two-point to five-point Likert scales, and one open question [31, 35]. In the present study the modules of depression (PHQ-9) with nine items (score range 0–27), somatic factors (PHQ-15) with 15 items (score range 0–30) and stress (PHQ-stress) with ten items (score range 0–20) were used [36, 37]. Higher scores indicate more severe symptoms [35–37].

As categories (cut-off scores) were used for PHQ-9: no-minimal (0–4), mild (5–9), moderate (10–14) moderate to severe (15–19) and severe (20–27); for PHQ-15: no-minimal (0–4), low (5–9), medium (10–14) and high (15–30), and for PHQ-stress: no-minimal (0–4), mild (5–9), moderate (10–14) and severe (15–20). Depression, somatisation and psychosocial stress were assumed for scores ≥5.

The SGRQ was designed to measure disease-specific health impairment in patients with chronic airflow limitation. It consists of two parts and measures three components (symptoms, activity and impact) and a total score each with a score range of 0–100. Higher scores indicate more limitations [32].

The STAI was developed to reveal state and trait anxiety and is used in clinical and research settings. Two forms are used, (X1-state (STAI-state) anxiety and *X*2-trait (STAI-trait) anxiety), with 20 items each and a four-point Likert scale. The scores can range from 20 to 80; higher scores mean more anxiety [33].

### Study approval

The study was approved by the ethics review committee of the University of Regensburg, Germany (approval number 09/097). Written informed consent was obtained from the patients.

### Statistical analysis

Continuous variables are shown as median values and quartiles. Spearman’s rank correlation coefficient was used to identify and measure the strength of the relationship between biomarkers. Associations between biomarkers and mental health were analysed using univariate linear regression analyses, with mental health as a dependent variable. Independent associations were assessed in an adjusted multivariate linear analysis (method: enter). Survival (until death from any cause) was analysed using the Mann–Whitney *U* test, Cox proportional hazard regression (univariate and, if applicable, multivariate) analyses, and Kaplan-Meier analyses (logrank test), starting by the day of inclusion and ending after 3-years of follow up. As cut-off we used median values. Variables significant in univariate Cox proportional hazard regression analyses and known risk factors for survival in COPD were entered into multivariate Cox proportional hazard regression analyses (method: stepwise backward likelihood ratio; probability for stepwise entry/removal: 0.05/0.10) to identify independent predictors. P <0.05 were considered statistically significant. SPSS version 20.0 (IBM SPSS Statistics, USA) was used for the data analysis.

## Results

### Study population

**Fig. 1:**
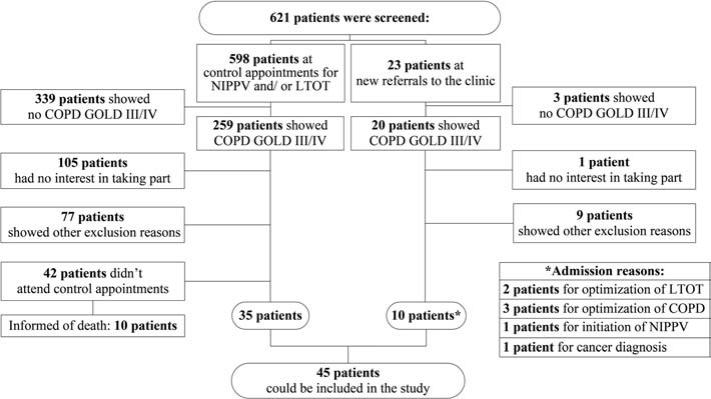
Patients’ recruitment. Notes: Other exclusion reasons: acute pneumonia confirmed by X-ray; lung resection; thoracic deformations; C-reactive protein (CRP) ≥20 mg/dl; blood pH <7.35; antibiotic therapy within the last 2 weeks. Abbreviations: NIPPV, non-invasive intermittent positive pressure ventilation; LTOT, long-term oxygen therapy

At the screening 279 patients out of 621 (44.9 %) showed GOLD stage III/IV. Among the 279 COPD GOLD stage III/IV patients, 106 (38 %) had no interest in participating, 86 (30.8 %) showed characteristics leading to exclusion (exacerbations, acute pneumonia, antibiotic therapy in the last 2 weeks, lung resection, thoracic deformations, or neuromuscular disease), and 42 (15.1 %) did not come to a scheduled visit (Fig. 1). Forty-five patients were eligible for inclusion in the study, and their characteristics are shown in Tables 1 and 2. No significant differences in patient characteristics were observed with respect to gender, with the exception of nicotine consumption, which was lower in females (32 (30; 50) *vs.* 53 (40; 80) pack/years; *p* = 0.024).

**Table 1:** Patient’s characteristics (*n* = 45)

Demographics	
Age, years	64 (57; 72)
Male	31 (68.9)
Female	14 (31.1)
BMI, kg/m^2^	28.1 (6.8)
Smoking history	
Active tobacco exposure, pack/years	50 (30; 70)
Current smoker	6 (13.3)
Ex-smoker	38 (84.4)
Never smoker	1 (2.2)
Spirometery	
VC, % predicted	61.8 (50; 71.4)
FEV_1_, % predicted	29.8 (22.6; 41.4)
FEV_1_/FVC, %	45.5 (39; 53.3)
Blood gas analysis^a^	
paO_2_, mmHg	62 (52.5; 73)
paCO_2_, mmHg	44 (40; 53.5)
BE, (mmol/l)	3.6 (1.45; 6.55)
pH	7.41 (7.39; 7.44)
mMRC dyspnea scale^b^	
Grade 0	3 (8.1)
Grade 1	1 (2.7)
Grade 2	5 (13.5)
Grade 3	9 (24.3)
Grade 4	19 (51.4)
Comorbidity- CIRS-G	
Total score^c^ (0-56)	13 (10; 17)
Severity index^d^ (0-14)	2.4 (2.2; 2.7)
NIPPV/LTOT	
NIPPV	35 (77.8)
LTOT	41 (91.1)
LTOT and NIPPV	33 (73.3)
Only LTOT	8 (17.8)
Only NIPPV	2 (4.4)
Medications (selection)	
Systemic corticosteroids^e^	15 (33.3)
Inhaled corticosteroids	37 (82.2)
LABA	37 (82.3)
SAMA	40 (88.9)
SABA and/or SAMA	31 (68.9)
Theophylline	18 (40)
Anxiolytics and/or antidepressants	19 (42.2)
Statins	13 (28.9)
Osteoporosis prophylaxis	17 (37.8)

*BMI* body mass index, CIRS-G Cumulative Illness Rating Scale-Geriatric, FEV1, forced expiratory volume in I second, *FVC* forced vital capacity, *LABA* long-acting beta-2 agonists, *LAMA* long-acting muscarinic antagonists, *LTOT* long-term oxygen therapy, *NIPPV* non-invasive intermittent positive pressure ventilation, *SABA* short-acting beta-2 agonists, *SAMA* short-acting muscarinic antagonists, *VC* vital capacity

Data are expressed as the median (quartile) for continuous variables and as frequencies (percentage) for categorical variables. ^a^with a mean (±SD) oxygen flow of 1.23 (±1.47) litres/min. ^b^ *n* = 37 due to poor patient compliance. ^c^sum of the 14 or system disease items (each with a five-point Likert scales). ^d^quotient of total score and total number of items endorsed. ^e^with a median (quartile) usage of 10 (5; 15) mg prednisolone daily

**Table 2:** Characteristics of questionnaires and serological biomarkers

Explanatory Variables	n	Median (quartiles)
Questionnaires:		
HADS (0-21)		
Anxiety	34	10 (8.75; 11)
Depression	34	8.5 (4; 11.3)
PHQ		
Depression (0-27)	33	6 (3; 11)
Somatisation (0-30)	33	10 (6; 15)
Stress (0-20)	33	6 (3; 8)
SGRQ (0-100)		
Total score	35	71.3 (57.5; 78.2)
Activity	35	85.9 (76.1; 92.5)
Impact	35	63.7 (42.5; 71.1)
Symptoms	35	69.2 (49; 76)
STAI (20-80)		
State anxiety	33	45 (34,5; 54)
Trait anxiety	33	45 (36; 49,5)
Biomarkers:		
Systemic inflammation:		
25-OHD (nmol/l)	44	38.4 (25.3; 66.5)
CRP (mg/dl)	45	2.6 (1.1; 8.7)
ESR (mm/h)	33	17 (7.5; 40)
Leucocytes (10^3^/μl)	45	9.7 (7.4; 12.4)
SA-A (mg/l)	43	8.1 (4; 17.8)
Cardiac dysfunction:		
NT-proBNP (pg/ml)	45	176 (65; 300)
Troponin I (ng/ml)	45	0.013 (0.007; 0.02)
Other:		
HbAIc (%)	45	6.2 (5.8; 6.9)
Hb (g/dl)	45	13.8 (13; 15.2)
Hc (%)	45	43 (40; 47)
Creatinine (mg/dl)	45	1.0 (0.8; 1.3)
TSH (mU/l)	45	0.8 (0.5; 1.2)

HADS cut-off scores: no (0-7), borderline (8-10), severe (11-14) and very severe (15-21) anxiety (HADS-anxiety) or depression (HADS-depression). PHQ-9 cut-off scores: no-minimal (0-4), mild (5-9), moderate (10-14) moderate to severe (15-19) and severe (20-27) depression. PHQ-15 cut-off scores: no-minimal (0-4), low (5-9), medium (10-14) and high (15-30) somatic factors. PHQ-stress cut-off scores: no-minimal (0-4), mild (5-9), moderate (10-14) and severe (15-20) psychosocial stress. For PHQ: Depression, somatisation and psychosocial stress were assumed for scores ≥5. SGRQ and STAI: no commonly established cut-off values. For all questionnaires: Higher scores indicate more limitations or more severe symptoms

*25-OHD* 25-OH-cholecalciferol, *CRP* C-reactive protein, *ESR* erythrocyte sedimentation rate, *HADS* Hospital Anxiety and Depression Scale, *HbAlc* glycosylated haemoglobin, *Hb* haemoglobin, *Hc* haematocrit, *NT-proBNP* N-terminal pro-brain natriuretic peptide, *PHQ* Patient Health Questionnaire,*SA-A* serum amyloid-A, *SGRQ* St. George’s Respiratory Questionnaire, *STAI* State-Trait Anxiety Inventory, *TSH* thyroid-stimulating hormone

### Mental health

Not all patients completed the questionnaires sufficiently (completed questionnaires: HADS, *n* = 34; PHQ, *n* = 33; STAI, *n* = 33; and SGRQ, *n* = 35). However, no significant differences were detected between patients who did or did not. Furthermore, no significant differences in mental health were found with regard to gender. The results of all questionnaires are shown in Table 2.

#### HADS: anxiety and depression

**Fig. 2:**
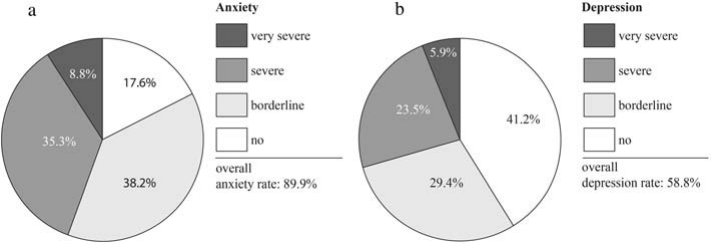
Hospital Anxiety (**a**) and Depression (**b**) Scale (HADS)

Anxiety, demonstrated by the HADS-A score, was present in 28 patients out of 34 (89.9 %); 13 patients out of 34 (38.2 %) showed borderline, 12 (35.3 %) severe, and 3 (8.8 %) very severe anxiety (Table 2, Fig. 2).

The prevalence of depression, demonstrated by the HADS-D score, was 20 patients out of 34 (58.8 %); 10 patients out of 34 (29.4 %) had borderline, 8 (23.5 %) severe, and 2 (5.9 %) had very severe depression.

#### PHQ: depression, somatisation and stress

**Fig. 3:**
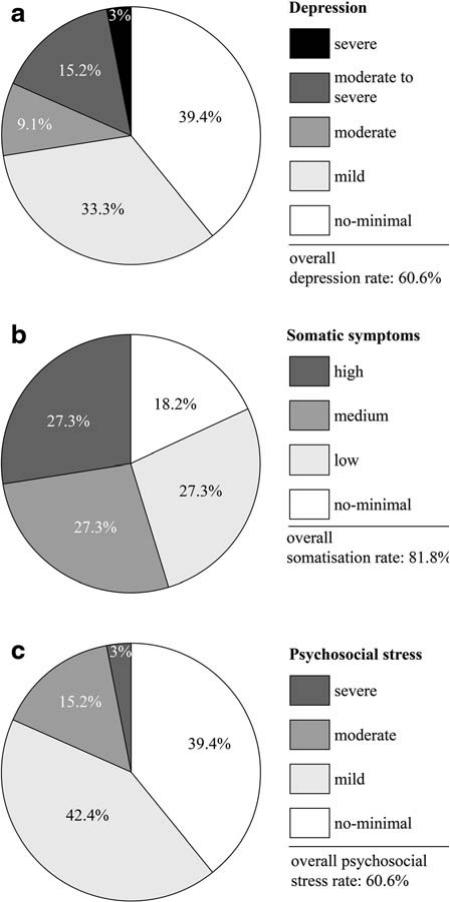
Patient Health Questionnaire (PHQ): scores of depression (**a**), somatic symptoms (**b**) and psychosocial stress (**c**)

The scores of PHQ-9 (Fig. 3) indicated a depression rate of 20 patients out of 33 (60.6 %). Mild depression was apparent in 11 patients out of 33 (33.3 %); moderate in 3 (9.1 %); moderate to severe in 5 (15.2 %) and severe depression in 1 (3 %). The somatisation rate, as demonstrated by PHQ-15 scores, was 27 patients out of 33 (81.8 %), with low, medium and high somatic symptoms split equally 9 patients out of 33 (27.3 %). The mild to severe stress rate, as demonstrated by PHQ-stress scores, was 20 patients out of 33 (60.6 %) with 14 patients out of 33 (42.4 %) mild cases, 5 (15.2 %) moderate and 1 (3 %) severe.

#### SGRQ and STAI

Due to the lack of commonly established cut-off values for SGRQ and STAI, only their median values are given in Table 2.

### Biomarkers

The results are presented in Table 2. SA-A was the only biomarker showing differences between genders. Female (*n* = 14) patients had higher values than male (*n* = 29) patients (16.7 (7.5; 61.4) mg/l *vs.* 7.3 (3.6; 9.0) mg/l; *p* = 0.039). Correlations between biomarkers are demonstrated in Tables 3 and 4.

**Table 3:** Spearman’s rank correlation coefficient between biomarkers I

	25-OHD (nmol/l)	CRP (mg-dl)	ESR (mm/h)	Leucocytes (10^3^/μl)	SA-A (mg/l)	NT-proBNP (pg/ml)
25-OHD (nmol/l)	1.00					
CRP (mg/dl)	0.08	1.00				
ESR (mm/h)	0.15	0.34	1.00			
Leucocytes (10^3^/μl)	0.15	0.18	0.07	1.00		
SA-A (mg/l)	0.17	0.512**	0.22	0.593**	1.00	
NT-proBNP (pg/ml)	0.06	0.14	−0.08	0.04	0.10	1.00
Troponin I (ng/ml)	0.00	0.12	0.10	0.18	0.11	0.493**
HbAlc (%)	−0.01	0.28	0.415*	0.18	0.11	0 07
Hb (g/dl)	−0.28	−0.25	−0.360*	0.02	−0.10	−0.05
Hc (%)	−0.355*	−0.19	−0.353*	0.06	−0.12	−0.04
Creatinine (mg/dl)	−0.26	−0.07	0.11	0.04	0.00	0.355*
TSH (mU/l)	−0.06	0.29	0.20	0.05	0.04	0.316*

*p = 0.05; **p = 0.01

*25-OHD* 25-OH-cholecalciferol, *CRP* C-reactive protein, *ESR* erythrocyte sedimentation rate, *SA-A* serum amyloid-A, *NT-proBNP* N-terminal pro-brain natriuretic peptide, *HbAlc* glycosylated haemoglobin, *Hb* haemoglobin, *Hc* haematocrit, *TSH* thyroid-stimulating hormone

Highlighted in bold: p ≤ 0.05

**Table 4:** Spearman’s rank correlation coefficient between biomarkers II

	Troponin I (ng/ml)	HbAlc (%)	Hb (g/dl)	Hc (%)	Creatinine (mg/dl)	TSH (mU/l)
Troponin I (ng/ml)	1.00					
HbAlc (%)	0.22	1.00				
Hb (g/dl)	0.12	0.24	1.00			
Hc (%)	0.19	0.27	0.946*	1.00		
Creatinine (mg/dl)	0.432*	0.25	0.07	0.13	1.00	
TSH (mU/l)	−0.01	0.08	−0.19	−0.20	0.06	1.00

Notes: *p = 0.01

*HbAlc* glycosylated haemoglobin, *Hb* haemoglobin, *Hc* haematocrit, *TSH* thyroid-stimulating hormone

Highlighted in bold: p ≤ 0.05

### Associations between mental health and serum biomarkers

In univariate linear regression analyses, there were inverse associations between depression, demonstrated by the HADS-D score, and the values for Hb (*p* = 0.041) or the Hc (*p* = 0.031). Positive associations were found between the PHQ-stress score and leucocyte numbers (*p* = 0.043), as well as between the STAI-trait anxiety score and leucocyte numbers (*p* = 0.03) or SA-A (*p* = 0.005) (Table 5). Because the Spearman’s rank correlation was high between the significant biomarkers, they were not included in a multivariate regression analysis.

**Table 5:** Linear univariate regression analysis of the questionnaire dimensions

Explanatory Variable	B Slope (95 % CI)	p*
Univariate analyses		
HADS anxiety		
Age (years)	−0.11 (-0.217-(-0.002))	0.045
VC (%)	−0.06 (-0.104-(-0.015))	0.01
FEV_1_ (%)	−0.1 (-0.174-(-0.02))	0.015
6-MWD (%)	−0.09 (-0.136-(-0.035))	0.002
RV/ TLC	0.042 (0.012-0.072)	0.007
HADS depression		
Hb (g/dl)	−0.94 (-1.838-(-0.041))	0.041
Hc (%)	−0.3 (-0.577-(-0.029))	0.031
6-MWD (%)	−0.12 (-0.234-(-0.012))	0.031
PHQ-15 (somatisation)		
6-MWD (%)	−0.15 (-0.276-(-0.025))	0.021
PHQ-stress		
Leucocytes (10^3^/μl)	0.4 (0.013-0.777)	0.043
SGRQ-total		
6-MWD	−0.44 (-0.714-(-0.166))	0.003
STAI-state anxiety		
VC (%)	−0.23 (-0.453-(-0.01))	0.041
STAI-trait anxiety		
Leucocytes (10^3^/μl)	1.1 (0.1117-2.089)	0.03
SA-A (mg/l)	0.17 (0.055-0.276)	0.005

*Only the significant (*p* ≤ 0.05) variables are shown. The questionnaire dimensions were the dependent variables

6-MWD, six-minute walk test, *ESR* erythrocyte sedimentation rate, FEV1, forced expiratory volume in 1 s, *Hb* haemoglobin, *Hc* haematocrit, *SA-A* serum amyloid-A, *RV* residual volume, *TLC* total lung capacity, *VC* vital capacity, *HADS* Hospital Anxiety and Depression Scale, *PHQ* Patient Health Questionnaire, *SGRQ* St. George’s Respiratory Questinnaire, *STAI* State-trait anxiety inventory

### Associations between mental health and functional indices

Inverse associations were demonstrated between the HADS-A score and VC (*p* = 0.01) and FEV_1_ (*p* = 0.015), and between STAI-state anxiety and VC (*p* = 0.041). Furthermore, positive associations were found between the HADS-A score and residual volume/ total lung capacity (*p* = 0.007). The 6-MWD score was negatively linked to several aspects of mental health: HADS-A (*p* = 0.002), HADS-D (*p* = 0.031), somatisation demonstrated by the PHQ-15 score (*p* = 0.021) and quality of life demonstrated by the total score of the SGRQ (*p* = 0.003). There was also an inverse association between the HADS-A score and age (*p* = 0.045) (Table 5). In multivariate linear regression analyses including significant biomarkers and functional indices, the 6-MWD score remained significant for HADS-A (*p* ≤ 0.024) and HADS-D (*p* = 0.042) (Table 6).

**Table 6:** Multivariate linear regression analysis of the questionnaire dimensios

Explanatory Variable	Slope (95 % Cl)	*p*	Model R^2^
HADS Anxiety			
RV/TLC	0.02 (-0.017-0.056)	0.272	
6-MWD (%)	−0.072 (-0.128-(-0.016))	**0.014**	0.401
VC (%)	−0.027 (-0.090-0.037)	0.391	
6-MWD (%)	−0.078 (-0.132-(-0.024))	**0.024**	0.394
FEV_1_ (%)	−0.079 (-0.124-0.065)	0.519	
6-MWD (%)	−0.030 (-0.135-(-0.024))	**0.008**	0.384
HADS Depression			
6-MWD (%)	−0.111 (-0.227-0.005)	0.059	
Hc (%)	−0.168 (-0.600-0.265)	0.429	0.228
6-MWD (%)	−0.111 (-0.228-(-0.005))	**0.042**	
Hb (g/dl)	−0.635 (-1.917-0.646)	0.314	0.243

*25-OHD* 25-OH-cholecalciferol, *6-MWD* six-minute walk test, *ESR* erythrocyte sedimentation rate, *FEV1*_*1*_ forced expiratory volume in 1 s, *Hb* haemoglobin, *Hc* haematocrit, *RV* residual volume, *SA-A* serum amyloid-A, *TLC* total lung capacity, *VC* vital capacity, *HADS* Hospital Anxiety and Depression Scale, *STAI* State-trait anxiety inventory. Questionnaire dimensions were the dependent variables

Highlighted in bold: p ≤ 0.05

### Survival

Each patient was observed for 3 years. Within this period 18 patients out of 45 (40 %) died; 9 patients (50 %) died of respiratory, 2 (11.1 %) of non-respiratory, and 7 (38.9 %) of not further specified causes.

**Fig. 4:**
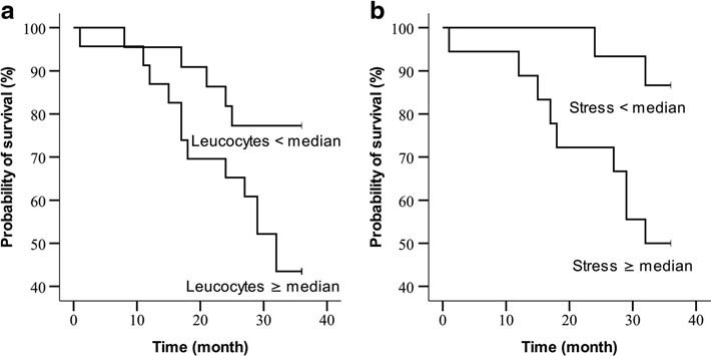
Kaplan Meier charts for leucocyte numbers (**a**) and PHQ-stress (**b**) using the respective median values as cut-off (leucocytes: median = 9.7 10^3^/μl, *p* = 0.028; PHQ-stress: median = 6, *p* = 0.023). PHQ, Patient Health Questionnaire; SGRQ, St. George’s Respiratory Questionnaire; STAI, State-Trait Anxiety Inventory

The Mann–Whitney-*U* test did not demonstrate any significant differences in the tested variables between survivors and non-survivors. However, using the median as cut-off in a Cox proportional hazard regression model, leucocyte numbers (hazard ratio (HR) 2.97, 95 % CI 1.06–8.36; *p* = 0.038) and PHQ-stress (HR 4.92, 95 % CI 1.06–22.85; *p* = 0.042) were significantly linked to long-term survival (Table 7 and Fig. 4). In multivariate Cox regression analyses including known risk factors for survival in COPD (6-MWD, body mass index, FEV_1_ (% predicted)) as well as PHQ-stress (model 1) and leucocytes (model 2), only PHQ-stress (HR 45.63, 95 % CI 1.72–1,208.48; *p* = 0.022) remained significantly related to survival, whereas leucocyte numbers did not (Table 7, model 1 and 2).

**Table 7:** Cox regression analysis of survival according to categorisation of variables < *vs.* > the median value

Explanatory Variable	HR (95 % CI)	*p
Univariate analysis		
PHQ-stress	4.92 (1.06-22.85)	**0.042**
Leucocytes	2.97 (1.06-8.36)	**0.038**
6-MWD (%)	2.07 (0.6-7.07)	0.248
BMI	0.77 (0.30-1.95)	0.58
FEV_1_, % predicted	1.51 (0.59-3.89)	0.395
Multivariate analyses		
Model I		
6-MWD (%)	19.58 (1.01-380.75)	**0.049**
BMI	48.73 (1.21-1969.65)	**0.039**
FEV_1_, % predicted	3.37 (0.35-32.55)	0.295
PHQ-stress	45.63 (1.72-1,208.48)	**0.022**
Model 2		
6-MWD (%)	2.6 (0.71-9.57)	0.151
BMI	1.34 (0.38-4.79)	0.650
FEV_1_, % predicted	1.03 (0.25-4.18)	0.970
Leucocytes	3.41 (0.91-12.85)	0.069

*Only the significant (*p* ≤ 0.05) univariate variables for physical function, demographic data and biomarkers are shown. Model 1 and 2: Cox proportional hazard model (method: stepwise backward likelihood ratio) including known risk factors for survival in COPD (6-MWD, body mass index, FEV_1_, (% predicted)) as well as PHQ-stress (model 1) and leucocytes (model 2)

*PHQ* Patient Health Questionnaire, *&-MWD* six-minute walk test, *BMI* body mass index, FEV1 forced expiratory volume in 1 s

Highlighted in bold: p ≤ 0.05

## Discussion

In this prospective study of patients with stable COPD of GOLD stages III and IV we have examined the association between mental health measures and serum biomarkers by various statistical methods. Biomarkers and mental health were also analysed for associations with survival. There were statistically significant but rather weak associations between some aspects of mental health and SA-A, Hb, Hc and leucocyte number. Among the mental health parameters analysed PHQ-stress and among the biomarkers only leucocyte number were associated with survival, whereas in an adjusted multivariate analysis only PHQ-stress remained as independent prognostic factor.

### Mental health

We found that the prevalence of anxiety (HADS-A: 84.9 %) and depression (HADS-D: 58.8 %; PHQ-9: 60.6 %) was higher than reported in studies that included patients in primary care with moderate to very severe COPD of comparable age (Centers for Epidemiologic Studies Depression scale (severe COPD): 25 % [38]; HADS-A (cut-off ≥11; moderate to very severe COPD): 32.7 %, HADS-D: (cut-off ≥11; moderate to very severe COPD): 20.8 % [39]). This was probably due to the selection, in our study, of patients with severe and very severe COPD, and the inclusion of patients with borderline anxiety and depression (HADS). Moreover, there were also differences in the questionnaires used. When we applied to our cohort the same HADS cut-off value as used in previous reports (cut-off ≥11), the prevalence of anxiety (46.7 %) and depression (29.4 %) was still higher. The exclusion of patients categorised with mild depression in the PHQ-9 (cut-off ≥10) gave a similar depression rate of 27.3 %. The frequency of somatisation (PHQ-15: 81.8 %) was also high in our study participants in comparison to the general population (PHQ-15: 9.3 %) [40]. The stress score was also higher compared to primary care patients of similar age (PHQ-stress score: 6 *vs.* 2.3) [41] and showed an increased stress rate (cut-off: ≥5) with 60.6 %. These results highlight the importance of mental health and psychiatric diagnoses particularly in patients with severe COPD, including the view that COPD has to be recognized as systemic disease that obviously has tremendous effects beyond the somatic dimension.

### Mental health and biomarkers

In our study, leucocyte number and the level of SA-A, biomarkers particularly related to systemic inflammation, were significantly correlated with mental health. In line with this finding, Justo and co-workers described associations between a history of mental crisis and inflammatory markers (fibrinogen and leucocyte number) in men [42]. Other investigators have reported associations between inflammation (defined by increased levels of cytokines and CRP) and neuropsychiatric disease, such as major depressive disease [43], generalised anxiety disorder [44], or even anxiety in healthy adults [45]. In accordance with this, Thompsen et al. demonstrated that elevated leukocytes levels are associated with a higher risk for comorbidities in COPD [46]. Prolonged neuroinflammation, induced by pro-inflammatory cytokines, is considered to play an important role in the development of neurobehavioral impairment, dementia and cognitive decline [29]. The inflamed brain microenvironment apparently leads to an overactivation of microglial cells with increased release of reactive oxygen species that cause neurotoxicity [47], and to dysfunction of central nervous system stem cells with resulting impairment in tissue homeostasis and repair function [48].

These hypotheses has been recently supported by the findings of Al-shair et al. [49] who described a strong positive correlation between tumor necrosis factor-alpha (TNF-α) and depression - measured by the Brief Assessment Schedule Depression Cards (BASDEC) - in mainly moderate and stable COPD patients [49]. TNF-α is an inflammatory protein that has been shown to be increased in stable COPD (compared to healthy control groups) and seems to increase even more in acute exacerbation of COPD [50]. As inflammatory mediator, besides others, it induces acute phase proteins including SA-A, which has been shown to be a more sensitive marker of an acute exacerbation of COPD than CRP alone or with dyspnea [51]. According to the present study, SA-A seems to be also a sensitive marker for negative effects on mental health in patients with stable and severe COPD.

In addition, in our study lower Hb and Hc levels were linked to higher depression values. Accordingly, we found in a large retrospective analysis that in patients with chronic respiratory failure, irrespective of the underlying aetiology, anaemia was independently related to dyspnoea and to a low HRQOL score when measured by the Severe Respiratory Insufficiency questionnaire [52]. Similar data were obtained by Cote and co-workers who investigated 683 stable COPD out-patients and found that anaemic patients showed a significantly higher modified Medical Research Council dyspnoea scale than non-anaemic patients [53]. In a *post-hoc* analysis of patients with COPD the physical function scores of the HRQOL questionnaire Short Form-36 were significantly lower in patients with anaemia compared to those without [54].

### Associations between mental health and functional data

We found significant negative correlations between 6-MWD values and several dimensions of mental health. The 6-MWD test is considered as an integrative measure influenced by physiological, physical and emotional aspects [11], and there is evidence that HRQOL increases with physical activity [55]. From this point of view, our findings regarding 6-MWD appear to be consistent with those of previous studies [56, 57].

Anxiety showed a negative linear association with age. Cleland et al. found a similar association with significantly higher anxiety and depression levels in COPD patients below the age of 60 years [39]. This was interpreted on the one hand due to a better acceptance of symptoms in the elderly as a predictable late life stressor, and on the other hand as an indication that in contrast to healthy subjects of the same age younger COPD patients have to deal with functional and physical impairment and therefore suffer more psychologically [39].

### Survival

Mental health in terms of PHQ-stress and systemic inflammation in terms of leucocyte levels were significantly related to 3-year survival in univariate survival analysis. Regarding the predictive value of leukocytes levels for long-term survival it appears reassuring that in the large Evaluation of COPD Longitudinally to Identify Predictive Surrogate Endpoints (ECLIPSE) study, comprising 1843 COPD patients, leukocyte numbers improved the predictive value of established risk factors [16]. In contrast, in the present investigation the level of serum CRP was not significantly linked to survival. This is in line with the results of the study of de Torres and co-workers who postulated that, particularly in patients with moderate to severe COPD, the magnitude of CRP-levels could be critically influenced by other factors such as morbidity (cardiovascular disease, metabolic syndrome, hypertension etc.), degree of physical activity, diet, smoking status and medications [58]. Conversely, the prognostic value of CRP was higher in large epidemiological studies covering patients with less severe COPD [59].

In the present study, neither depression, nor anxiety has shown to be associated with survival. Other studies revealed depression as negative prognostic factor for mid- to long-term survival (1 to 3 years) for COPD patients being in a stable state of disease [6–8], and for short-term survival (6 months) after recovering from an acute exacerbation [60]. Possible reasons for this differences might be the use of different cut-off values, questionnaires and observation time (Beck Depression Inventory: cut-off ≥19 [8], quintile of ≥15 compared with <5 [7]; Yesavage depression score: cut-off 5 and 11 [6]; HADS-A/D: cut-off ≥8 [60]). Furthermore, anxiety was also no significant predictor of mid- to long-term survival [7], but marginal significant after acute exacerbation [60].

To the best of our knowledge, this is the first study to investigate the PHQ-stress score and survival in patients with COPD. Psychosocial stress occurs when the individual recognises that environmental demands strain or exceed their adaptive capability. Chronic psychosocial stress in particular seems to influence the course of diseases, including cardiovascular disease, upper respiratory infections, autoimmune diseases, diabetes and depression [61]. This is in line with our findings of an association of increased psychosocial stress levels and mortality in severe COPD. Moreover, sleep is a vital counterbalance to stress enabling the body to recover and is essential for coping with stress [62]. Therefore, a disease such as COPD, with its high prevalence of sleep disturbance, is at risk of causing increased stress. Chronic psychosocial stress also can lead to glucocorticoid receptor resistance and consequently interferes with the control of inflammation [63], which could also explain its positive linear correlation with leucocyte numbers.

### Limitations

Firstly, the small sample size allowed detecting only relatively strong associations, and limited statistical power especially for multivariate analyses. Secondly, the patients’ motivation to start and to complete the questionnaires apparently dwindled with the number of pages. Fewer questionnaires would have increased motivation and compliance. Thirdly, not all questionnaires have an accepted score classification; therefore, the use of median values was the simplest way for comparison. It is, therefore, possible that associations would be different using other classification systems. Fourthly, corticosteroids (systemic and/or inhaled) and statins might influence especially inflammation markers like CRP, leucocyte numbers and SA-A; osteoporosis prophylaxis might affect the level of 25-OHD, and anxiolytics, antidepressants, morphine/morphine derivates, corticosteroids and theophylline the questionnaire results. Finally, the seemingly paradoxical tendencies regarding survival and lung function or 6-MWD suggest that we had to deal with a specific selection of patients. In view of this it seems of even greater interest that psychosocial stress was a predictor for survival even in patients with a tendency towards prolonged survival despite worse lung function or 6-MWD.

## Conclusion

In conclusion, the different dimensions of mental health assessed in this pilot study were related to serum biomarkers although these associations were rather weak. This might be indicative of the heterogeneity of the disease. Regarding the predictive value for long-term survival, the PHQ-stress score was an independent prognostic factor in multivariate analyses, whereas among biomarkers, only leukocyte numbers showed an association and only in univariate analysis. The observation that psychosocial stress had a greater prognostic value than conventional biomarkers suggests that this easily measured parameter would be useful in the prognostic assessment of patients with severe COPD and – with regard to the multiple systemic effects - probably should be included in the assessment of COPD.

This pilot study may help to design further larger prospective trials to investigate the associations between mental health, serum biomarkers and survival in severe COPD in more detail.
